# A three miRNAs panel in paraffin tissue serves as tool for predicting prognosis of renal cell carcinoma

**DOI:** 10.3389/fonc.2024.1391844

**Published:** 2024-04-24

**Authors:** Wenkang Chen, Wuping Wang, Zhengping Zhao, Zhenyu Wen, Yingqi Li, Zhenjian Ge, Yongqing Lai, Liangchao Ni

**Affiliations:** ^1^ Guangdong and Shenzhen Key Laboratory of Reproductive Medicine and Genetics, Department of Urology, Peking University Shenzhen Hospital, Shenzhen, China; ^2^ Shantou University Medical College, Shantou, Guangdong, China; ^3^ Shenzhen University Medical College, Shenzhen, Guangdong, China

**Keywords:** biomarker, renal cell carcinoma, kidney cancer, cancer diagnosis, miRNA, paraffin tissue

## Abstract

**Background:**

Renal cell carcinoma (RCC) stands as the most prevalent form of urogenital cancer. However, there is currently no universally accepted method for predicting the prognosis of RCC. MiRNA holds great potential as a prognostic biomarker for RCC.

**Methods:**

A total of 100 cases with complete paraffin specimens and over 5-year follow-up data meeting the requirements were collected. Utilizing the clinical information and follow-up data of the specimens, an information model was developed. The expression levels of eight microRNAs were identified using RT-qPCR. Finally, determine and analyze the clinical application value of these microRNAs as prognostic markers for RCC.

**Results:**

Significant differences were observed in the expression of two types of miRNAs (miR-378a-5p, miR-23a-5p) in RCC tissue, and three types of miRNAs (miR-378a-5p, miR-642a-5p, miR-23a-5p) were found to be linked to the prognosis of RCC. Establish biomarker combinations of miR-378a-5p, miR-642a-5p, and miR-23a-5p to evaluate RCC prognosis.

**Conclusion:**

The combination of three microRNA groups (miR-378a-5p, miR-642a-5p, and miR-23a-5p) identified in paraffin section specimens of RCC in this study holds significant potential as biomarkers for assessing RCC prognosis.

## Introduction

In 2020, there were approximately 431288 newly diagnosed instances of kidney cancer (KC) worldwide, with the death toll reaching 179368 (1). Histologically, kidney cancer (KC) cases are overwhelmingly attributed to RCC, which accounts for 90% of all cases (2).. Arising from the nephron, renal cell carcinomas (RCC) form a diverse collection of malignant neoplasms with varying characteristics (3).. Early-stage cancer typically does not present with obvious symptoms, resulting in over 60% of patients being incidentally detected during routine ultrasound examinations (4, 5). Unfortunately, many patients are already in the advanced stage by the time they receive their initial diagnosis. Even after undergoing radical nephrectomy, about 30% of patients may still experience tumor recurrence (6). The survival rate is significantly influenced by the stage at which the diagnosis occurs. Localized disease, representing stage I, carries a 5-year relative survival rate of 93%. When the disease progresses to regional involvement (stage II/III), with local lymph nodes affected, the 5-year relative survival rate drops to 72.5%. However, when the disease reaches the metastatic stage (stage IV), the 5-year relative survival rate plummets to a mere 12% (7).. The earlier the prognosis of a patient is determined and treatment is initiated, the better the treatment outcome is expected to be. As a result, regular comprehensive CT scans are scheduled for patients to promptly detect tumor recurrence or metastasis to other areas following surgery, facilitating timely and effective treatment (8). However, frequent CT scans expose the patient’s body to long-term radiation imaging and impose burdens of cost and time loss (9, 10). Currently, there is no universally accepted standard in clinical practice for accurately evaluating the prognosis of renal cancer patients. Most evaluations rely on pathological staging and classification, which do not fully meet clinical needs (11, 12). Consequently, there is a requirement for a biomarker in clinical practice that can precisely forecast the prognosis of RCC. This would enable precise treatment tailored to individual prognoses and reduce the physical and financial burdens experienced by patients.

Ranging from 19 to 25 nucleotides (nt) in length, microRNAs (miRNAs) are noncoding RNA molecules with the ability to control gene expression at both transcriptional and translational levels (13, 14). They are powerful regulators of various cellular activities including cell growth, differentiation, development, and apoptosis (15). In cancer, miRNAs that are dysregulated are categorized into oncogenes (oncomiRs) or tumor suppressors. OncomiRs enhance and suppress their target tumor suppressor genes within cancer. Conversely, in malignant tumors, tumor suppressor miRNAs are reduced, leading to the increased expression of their target oncogenes. This suggests that inhibition or overexpression of miRNA may serve as a pathway for predicting tumor occurrence (16, 17). Compared to proteins and mRNA, miRNA exhibits stronger stability, ease of detection, reduced susceptibility to degradation, and cost-effectiveness (18). Furthermore, miRNA can be reliably extracted from cells, tissues, and body fluids (19). As a result, microRNAs in paraffin pathological sections have significant potential to serve as prognostic markers for renal cell carcinoma.

## Materials and methods

### Specimen collection

The 100 specimens were acquired from Peking University Shenzhen Hospital. The surgeries took place before 2015, and the follow-up data spans over 5 years. None of the participants underwent treatments such as chemotherapy or radiotherapy before their surgeries. All specimens were surgically removed and pathologically diagnosed as RCC, with a 2010 AJCC renal cancer TNM stage of ≥ II. Following resection, the specimens were promptly placed in RNA protective solution (Qiagen GmbH, Hilden, Germany), and then preserved in a refrigerator at -80°C. Permission for this subsequent information was secured from the Independent Ethics Committee of Peking University Shenzhen Hospital, and the supervision of the committee was adhered to during the subsequent procedures.

### Clinicopathological parameters of RCC patients

By collecting the hospitalization history of patients, we were able to gather clinical information such as age, gender, date of birth, operation date, tumor size, tumor stage, and the treatment received. In addition, we categorized the histology and tumor stage of RCC according to medical and pathological perspectives.

### Determination of miRNA expression

Adhering to the RNA extraction from paraffin protocol (miRNeasy FFPE Kit), the sample was deposited in a 1.5 ml centrifuge tube. Then, 160 μl of Deparaffinization Solution was added before the EP tube was incubated at 56°C in a water bath for 3 minutes. After reaching room temperature, 150 μl of Buffer PKD was introduced and blended via shaking, and then centrifuged at 4°C for 1 minute at 11,000g. Drawing from the lower segment of the EP tube with a pipette gun, 10 μl of Proteinase K was dispensed and the tube was then incubated at 56°C for 15 minutes before immediate transfer to an 80°C water bath for 15 minutes. Thereafter, the EP tube would be stratified and the colorless fluid from the bottom section transferred to a new EP tube, to be chilled on ice for 3 minutes and then centrifuged at 20,000 g for 15 minutes at 4°C. The newly retrieved supernatant was then relocated to a fresh microcentrifuge tube. 15 μl of DNase Booster Buffer and 10 μl of DNase I stock solution were subsequently added, followed by a short period of centrifugation and a 15-minute room temperature incubation. An additional 320 μl of Proteinase K was then added and after another brief round of centrifugation and room temperature incubation, 320 μl of Buffer RBC and 1120 μl of Anhydrous Ethanol (100%) were mixed in by pipetting. This was then transferred to a 2 ml collection tube without centrifugation and was centrifuged for 15 seconds at ≥8000 x g (≥10000 rpm). The precipitate in the collection tube was discarded and the process was repeated, ensuring the entire sample passed through the collection column. 500 μl of Buffer RPE was then added and the tube was agitated before a 15-second centrifugation at ≥8000 x g (≥10000 rpm). The residue was discarded. An RNeasy MinElute Spin Column was then placed in a new 2 ml collection tube and centrifuged at maximum speed for 5 minutes. The liquid was discarded and the RNeasy MinElute Spin Column placed in a new 1.5 ml collection tube. 14-30 μl of RNase-free water was then directly added to the spin column membrane which was then washed with a full-speed centrifuge for one minute.

In the following steps, a mixture was prepared by combining 1 μg of total RNA, 4 μl of 5× Hispec buffer, 2 μl of 10× Nuclecs Mix, and 2 μl of RT, with the volume adjusted to 20 μl using RNase-free water. This mixture was subsequently placed into a real-time quantitative PCR system (LightCycler® 480 Fluorescent Quantitative PCR System, Roche Diagnostics, Basel, Switzerland).The real-time quantitative PCR procedure commenced with an initial denaturation at 95°C for 15 minutes, succeeded by 40 rounds of 94°C for 15 s, 55°C for 30 s, and 72°C for 30 s. The 2−△△Cq method was employed to ascertain the relative expression levels of the target miRNAs. The primer information is shown in **Table 1**.

**Table 1:** Sequences of microRNAs primers.

microRNAs	Sequence
miR-23a-5p forward primer	5'-ggggttcctggggatg-3'
miR-23a-5p reverse primer	5'-caggtccagtttttttttttttttaaatc-3'
miR-181a-5p forward primer	5'- cattcaacgctgtcggt-3'
miR-181a-5p reverse primer	5'- ggtccagtttttttttttttttactca-3'
miR-487a-5p forward primer	5'-caggtggttatccctgct-3'
miR-487a-5p reverse primer	5'- tccagtttttttttttttttcgaaca-3'
miR-592 forward primer	5'-gcagttgtgtcaatatgcga-3'
miR-592 reverse primer	5'- ggtccagtttttttttttttttacatca-3'
miR-658 forward primer	5'-ggagggaagtaggtccgt-3'
miR-658 reverse primer	5'- ggtccagtttttttttttttttacca-3'
miR-33b-5p forward primer	5'-gcaggtgcattgctgt-3'
miR-33b-5p reverse primer	5'- gtccagtttttttttttttttgcaat-3'
miR-378a-5p forward primer	5'-tcctgactccaggtcct-3'
miR-378a-5p reverse primer	5'- ggtccagtttttttttttttttacaca-3'
miR-642a-5p forward primer	5'-gtccctctccaaatgtgtc-3'
miR-642a-5p reverse primer	5'- ggtccagtttttttttttttttcaag-3'

### Statistical analysis

For this study, SPSS 20.0 was utilized to analyze the data. We determined the optimal cutoff value by using the maximum Jordan index method based on the expression levels of different miRNAs. 2−△△Cq >1 indicates high expression, while 2−△△Cq method<1 indicates low expression. Model construction was performed for the collected clinical information of the patients. Independent samples t-test was conducted for the measurement data, and rank sum test was performed for the count data. Kaplan-Meier survival analysis was utilized for one-way analysis, with p < 0.05 denoting statistical significance.

## Result

### Expression levels of miRNA and clinicopathologic characteristics of patients

The relationship between the expression levels of different miRNAs and patients’ clinical characteristics was assessed using the chi-square test. As depicted in **Table 2**, the findings show no significant association between the miRNAs’ relative expression levels and the clinical features of the cases (p > 0.05).

**Table 2:** Clinical characteristics and miRNA expression.

Total	Variables	Age (Years)		Gender		Pathological grade		
		>60	≤0a	Male	Female	High	Low	
No. of miR-378a-5p (cases)								p>0.05
High	22	5	17	9	13	8	14	
Low	78	29	49	48	30	29	49	
miR-185a-5p								p>0.05
High	86	26	60	47	39	31	55	
Low	14	8	6	10	4	6	8	
miR-642a-5p								p>0.05
High	35	12	23	22	13	11	24	
Low	65	22	43	35	30	26	39	
miR-592								p>0.05
High	19	9	10	12	7	11	8	
Low	81	25	56	45	36	26	55	
miR-658								p>0.05
High	34	11	23	21	13	11	23	
Low	65	23	43	36	30	26	40	
miR-23a-5p								p>0.05
High	50	20	30	28	22	22	28	
Low	50	14	36	29	21	15	35	
miR-487a-3p								p>0.05
High	61	21	40	36	25	24	37	
Low	39	13	26	21	18	13	26	
miR-33b-5p								p>0.05
High	44	11	33	25	19	13	31	
Low	56	23	33	32	24	24	32	

### Expression level of miRNA between survival and death

**Figure 1:**
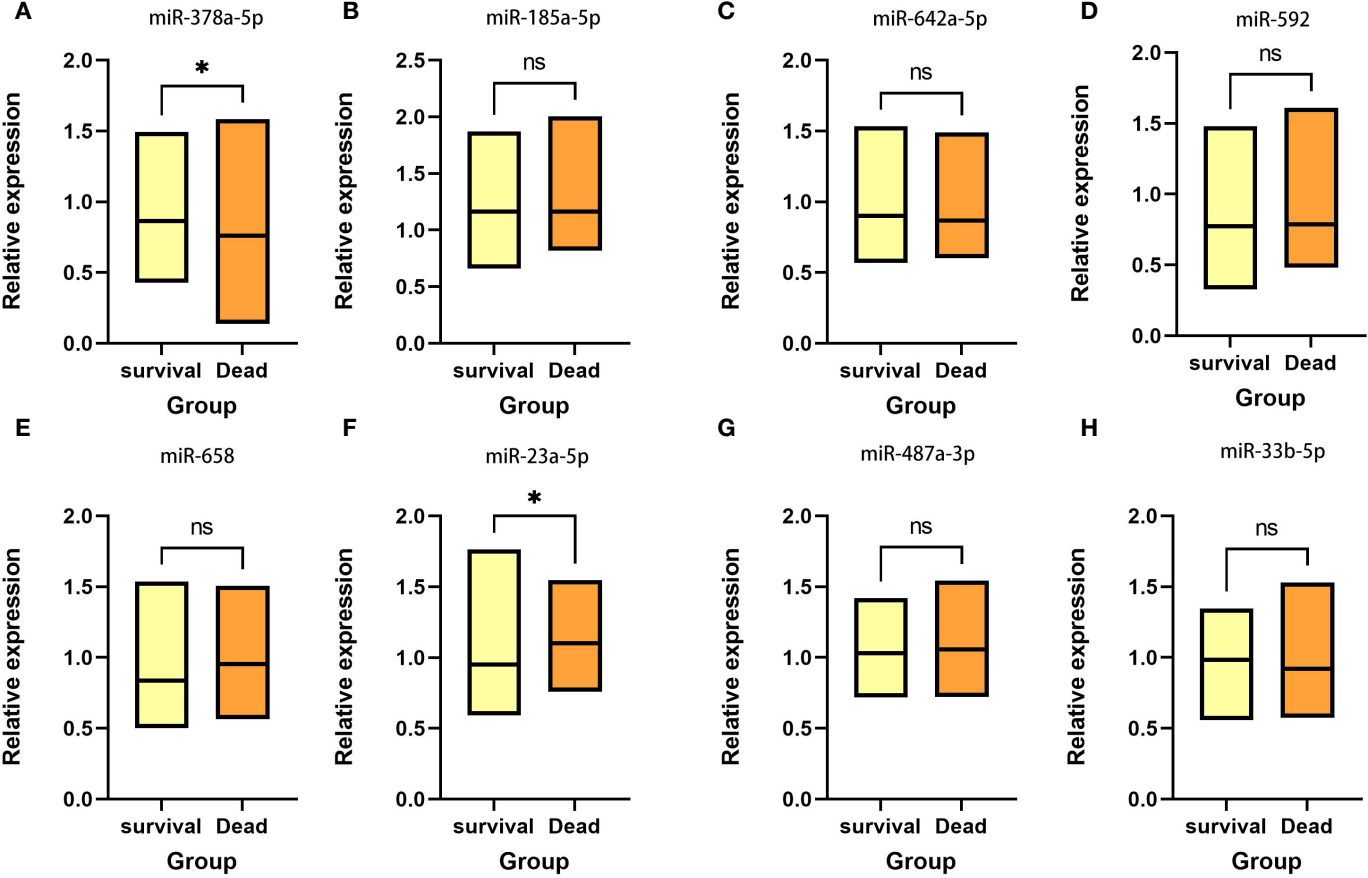
Expression between survival group and death group There is a significant elevation in the expression level of **(A)** miR-378a-5p within the survival group, whereas there is a considerable decrease in the expression level of **(F)** miR-23a-5p. No statistical significance was detected in **(B)** miR-185a-5p, **(C)** miR-642a-5p, **(D)** miR-592, **(E)** miR-658, **(G)** miR-487a-3p and **(H)** miR-33b-5p. *p < 0.05; ns, Non-significant.

Based on the five-year follow-up survival status, 65 individuals were classified into the survival group, while 35 individuals were categorized into the death group. **Figure 1** shows that in the survival group, the expression level of miR-378a-5p is markedly elevated (p<0.05), whereas the expression level of miR-23a-5p is significantly reduced (p<0.05).

### Prognostic correlation analysis of miR-378a-5p, miR-642a-5p, and miR-23a-5p in RCC

**Figure 2:**
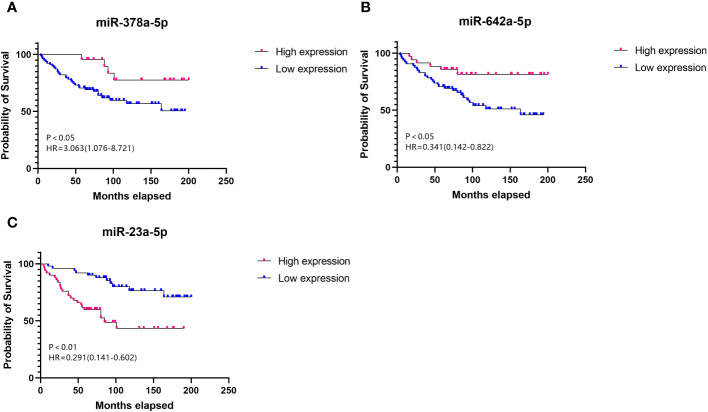
Survival curve Elevated levels of **(A)** miR-378a-5p and **(B)** miR-642a-5p, along with reduced levels of **(C)** miR-23a-5p, serve as prognostic protective factors for RCC.

Cox regression analysis indicated that higher expression levels of miR-378a-5p (**Figure 2A**, HR=3.063, 95% CI=1.076-8.721, p<0.05) and miR-642a-5p (**Figure 2B**, HR=0.341, 95% CI=0.142-0.822, p<0.05) correlate positively with the survival rates following surgery for RCC. In contrast, a negative correlation with postoperative survival was observed for the expression levels of miR-23a-5p (**Figure 2C**, HR=0.291, 95% CI=1.076-8.721, p<0.05). This suggests that increased levels of miR-378a-5p and miR-642a-5p are linked to longer overall survival, while higher expression levels of miR-23a-5p are correlated with shorter overall survival.

### A composite miRNA panel for the evaluation of RCC prognosis

**Figure 3:**
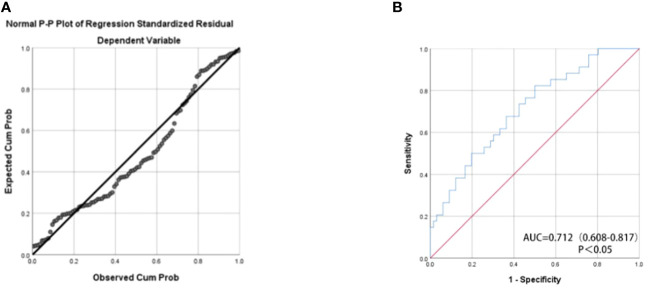
**(A)** Normal P-P Plot of Regression Standardized Residual The actual observed curve fits well with the predicted curve of the panel. **(B)** The receiver operating characteristic (ROC) curve analyses for the three miRNA panel (miR-378a-5p, miR-642a-5p, and miR-23a-5p).The combination of these three miRNAs has moderate predictive accuracy.

We assessed the predictive potential of various miRNAs for renal cancer prognosis, recognizing that combining multiple miRNAs may yield superior predictive performance compared to using a single miRNA. The formula for the final logistic regression model established is as follows: logit (P) = 104.33 + 41.153 x miR-378a-5p + 43.598 x miR-642a-5p - 82.576 x miR-23a-5p. As depicted in **Figure 3A**, the established equation exhibits a high degree of fitting and is deemed reliable for usage. Utilizing a panel of miR-378a-5p, miR-642a-5p, and miR-23a-5p as biomarkers (p<0.05) may be an effective approach for predicting the prognosis of RCC. As depicted in **Figure 3B**, the AUC value of the combination of these three miRNAs is 0.712 (p<0.05), indicating moderate accuracy in prediction.

## Discussion

Comprising 2-3% of all malignant tumors in adults, renal cell carcinoma (RCC) stands as the most common type of urogenital carcinoma. It is more frequently diagnosed in men than in women. Around 33% of RCC patients develop metastases, and the mortality rate can reach as high as 30-40% (7, 20, 21). This clearly demonstrates that RCC poses a significant risk to human life and well-being. The survival rate of RCC patients over a 5-year period varies significantly depend on their stages and grades. Accurately determining the prognosis is a crucial step in evaluating renal cell carcinoma patients. It can facilitate the initiation of new adjuvant therapies, such as the targeted drug sunitinib, and predict the progression of the disease (22). Given the limited efficacy of traditional chemotherapy or immunotherapy, a better understanding of prognosis can help patients select the optimal timing and methods of treatment, thereby maximizing treatment effectiveness and reducing the likelihood of recurrence or metastasis (23). This, in turn, can reduce the physical and economic burden caused by regular follow-up examinations. Regrettably, there is currently no gold standard in clinical practice that can accurately assess the prognosis of RCC (24). Consequently, there is an urgent need for effective biomarkers that can evaluate the prognosis of RCC in clinical practice. MiRNA in RCC issue has tremendous potential and is expected to become a biomarker that fulfills clinical requirements for predicting the prognosis of RCC.

The experimental findings suggest that miR-378a-5p, miR-642a-5p, and miR-23a-5p are correlated with the prognosis of renal cell carcinoma. Nevertheless, there is no noteworthy association between the case’s clinical characteristics and the relative expression levels (p>0.05). Furthermore, the combination of these miRNAs exhibits improved predictive performance and holds potential as a prognostic biomarker for RCC patients. MiRNA is a potent regulatory factor and has been demonstrated to drive carcinogenic pathways (25). There is a wealth of evidence indicating that miR-378a-5p is commonly downregulated in colorectal cancer, resulting in the suppression of cell proliferation and increased resistance to apoptosis. It also downregulates CDK1 levels, thereby inhibiting the development of colorectal cancer (26). In addition, targeting the VEGF pathway, miR-378a-5p has been discovered to enhance the prognosis and hinder the progression of hepatocellular carcinoma (27).. In prostate cancer, miR-642a-5p functions as a suppressor of tumor growth, effectively diminishing the proliferation of cells associated with prostate cancer (28).. Increased expression of miR-642a-5p has demonstrated a suppressive effect on the migratory and invasive capabilities of colon cancer cells. Conversely, decreased levels of miR-642a-5p in individuals with colon cancer are correlated with an unfavorable prognosis (29).. Additionally, miR-23a-5p has been identified to suppress the growth and invasion of pancreatic ductal adenocarcinoma cells through the inhibition of ECM1 expression (30). It is also involved in the growth inhibition of liver cancer induced by andrographolide (31). These investigations have demonstrated that a solitary miRNA possesses the ability to modulate numerous target genes, thereby exerting influence over a broad spectrum of biological functions (32).. Notably, these investigations have shed light on the tumor-suppressive impacts of miR-378a-5p, miR-642a-5p, and miR-23a-5p, highlighting their significant correlation with tumor prognosis. They also suggest the significant potential of miR-378a-5p, miR-642a-5p, and miR-23a-5p as biomarkers for evaluating the prognosis of renal cell carcinoma.

## Conclusions

In conclusion, the panel of miR-378a-5p, miR-642a-5p, and miR-23a-5p holds promise as a prognostic biomarker for renal cell carcinoma, addressing the pressing clinical needs. Although numerous studies have verified the suppressive effects of miR-378a-5p, miR-642a-5p, and miR-23a-5p on tumor growth and invasion, additional investigation is required to clarify their precise functions in the molecular pathways that contribute to the initiation and progression of renal cell carcinoma. Subsequent studies in this area can enhance their clinical value as prognostic markers for renal cell carcinoma.

## Data availability statement

Datasets are available on request: The raw data supporting the conclusions of this article will be made available by the authors, without undue reservation.

## Ethics statement

The studies involving humans were approved by Independent Ethics Committee of Peking University Shenzhen Hospital. The studies were conducted in accordance with the local legislation and institutional requirements. The participants provided their written informed consent to participate in this study.

## Author contributions

WC: Formal analysis, Validation, Visualization, Writing – original draft, Writing – review & editing. WW: Writing – original draft, Writing – review & editing. ZZ: Data curation, Writing – review & editing. ZW: Data curation, Writing – review & editing. YLi: Data curation, Writing – review & editing. ZG: Data curation, Writing – review & editing. YLa: Conceptualization, Writing – review & editing. LN: Conceptualization, Writing – review & editing.
